# Advances in the application and mechanism of bioactive peptides in the treatment of inflammation

**DOI:** 10.3389/fimmu.2024.1413179

**Published:** 2024-08-23

**Authors:** Haiyang Liu, Lulu Zhang, Jingmou Yu, Shengwen Shao

**Affiliations:** ^1^ Huzhou Key Laboratory of Medical and Environmental Applications Technologies, School of Life Sciences, Huzhou University, Huzhou, China; ^2^ Key Laboratory of Vector Biology and Pathogen Control of Zhejiang Province, Huzhou University, Huzhou, China

**Keywords:** bioactive peptides, inflammation, immunomodulation, inflammatory mediators, pathways

## Abstract

Inflammation is a normal immune response in organisms, but it often triggers chronic diseases such as colitis and arthritis. Currently, the most widely used anti-inflammatory drugs are non-steroidal anti-inflammatory drugs, albeit they are accompanied by various adverse effects such as hypertension and renal dysfunction. Bioactive peptides (BAPs) provide therapeutic benefits for inflammation and mitigate side effects. Herein, this review focuses on the therapeutic effects of various BAPs on inflammation in different body parts. Emphasis is placed on the immunomodulatory mechanisms of BAPs in treating inflammation, such as regulating the release of inflammatory mediators, modulating MAPK and NF-κB signaling pathways, and reducing oxidative stress reactions for immunomodulation. This review aims to provide a reference for the function, application, and anti-inflammation mechanisms of BAPs.

## 1 Introduction

Inflammation is a normal immune response of the body’s innate and adaptive immune systems to infections (1), which can protect the body from damage caused by external toxins and stimuli (2). It is a way to self-heal, repair damaged tissues, and combat pathogens (3). However, the attack of inflammatory factors will result in cellular necrosis and the reduction of metabolic and immune functions, eventually leading to tissue damage and organ dysfunction. The duration of inflammation is different, which could be divided into acute and chronic inflammation (4). Many chronic diseases are associated with inflammation, including arthritis, inflammatory bowel disease (5), cardiovascular diseases (6), osteoporosis (7), cancer (8), and obesity (9). Therefore, combating inflammatory damage is one of the major health challenges of the 21st century. Non-steroidal anti-inflammatory drugs (NSAIDs), such as aspirin and ibuprofen, are a class of chemically synthesized anti-inflammatory drugs that do not contain steroid structures (10). They are the most widely used anti-inflammatory drugs. However, numerous studies have shown that NSAIDs have various side effects on the host, including hypertension, nephrotic syndrome, cardiovascular toxicity, acute renal failure, and gastrointestinal complications (1). Additionally, antibiotics can be used to treat inflammation, but they can induce to the emergence of antibiotic-resistant superbugs. Therefore, there is an urgent need to explore new strategies for anti-inflammation. Since the first antimicrobial peptide Cecropins was discovered in 1981, the antibacterial and anti-inflammatory activity of peptides has attracted more and more attention from academia (11). Research has described that the peptide GPETAFLR possessed anti-inflammatory activity, effectively inhibiting neuroinflammation and maintaining stability in the central nervous system (12).

BAPs refer to short-chain amino acid sequences with active biological functions within organisms, typically consisting of 2 to 20 amino acid residues interconnected by peptide or amide bonds (13). The arrangement and combination of these amino acid residues are different and can form linear or cyclic structures (13). The sources of BAPs are diverse, mainly including animals, plants, microorganisms, marine organisms, soy products, milk, and fermented products (14). When BAPs remain inactive within parent proteins, they can become active upon enzymatic release through peptide cleavage (15). Apart from being generated through the hydrolysis of parent proteins, BAPs can also be produced via microbial fermentation. In order to obtain BAPs with specific activity, specific proteases with a wide range of functions are usually used for hydrolysis (16).

Peptides offer several advantages over traditional drugs in disease treatment (17). For example, their low molecular weight allows them to penetrate membranes effectively (18, 19), making them more potent (20). Furthermore, bioactive peptides (BAPs) have the potential for targeted therapy with minimal or negligible toxicity, even at low concentrations (21). Inflammation occurs after the activation of inflammatory pathways by triggering factors, leading to the release of inflammatory agents (22). Concurrently, the anti-inflammatory characteristics of BAPs may be influenced by molecular weight, amino acid composition (hydrophobic amino acids, positively charged amino acids, specific amino acids), and amino acid position (3).

This review provides a detailed overview of the research status of BAPs in the treatment of skin inflammation, intestinal inflammation, pulmonary inflammatory disease, arthritis, and ocular inflammation. Subsequently, it delves into the immunomodulatory mechanisms employed by BAPs in the treatment of inflammation, such as regulating the release of inflammatory mediators, modulating mitogen-activated protein kinase (MAPK) and nuclear factor κB (NF-κB) signaling pathways, and reducing oxidative stress response for immunomodulation. The aim is to seek new strategies for inflammation treatment and provide references for the development and application of anti-inflammatory peptides.

## 2 The functions of BAPs

BAPs exhibit a wide array of functions including antimicrobial, antioxidative, anti-inflammatory, memory-enhancing, antithrombotic and antihypertensive activities, regulation of gastrointestinal absorption, appetite suppression, opioid modulation, immune modulation, and cell regulation. According to different functions, BAPs are mainly divided into anti-inflammatory peptides, antimicrobial peptides (AMPs), antioxidant active peptides, anticancer active peptides, antihypertensive peptides, and neuropeptides (**Table 1**). Anti-inflammatory peptides can modulate immune responses and alleviate inflammation. They can suppress the production of pro-inflammatory cytokines and the activation of inflammatory pathways, or directly interact with immune cells. BAPs with antibacterial activity are called AMPs.The activity of AMPs may be attributed to their ability to effectively disrupt bacterial cell walls or membranes with a strong negative charge, exerting their action with cations and their hydrophobic effect (15). They may also attack microbial membranes or cytoplasmic components, altering their cellular functions and leading to cell death (23). AMPs can inhibit the synthesis of cell walls, nucleic acids, and proteins by engaging various enzymes within target cells (23). AMPs possess minimal to provoke resistance (24), thereby conferring a natural advantage over antibiotics for combating microbial infections. Han et al. (25) discovered that AMPs containing tryptophan can downregulate the expression of DNA replication initiation genes in cells, consequently demonstrating efficacy in combating multidrug-resistant *Pseudomonas aeruginosa*.

**Table 1:** The names/sequences and source of BAPs with different functions.

Species	Peptide names/sequences	Source	Reference
Anti-inflammatory peptides	GPETAFLR	*Lupinus angustifolius* L.	(34)
	DAPAPPSQLEHIRAA, AADGPMKGILGY	*Lateolabrax maculatus*	(35)
	SSEDIKE	Amaranth proteins	(36)
	Lectin	Red algae *Amansia multifida*	(37)
	VHYAGTVDY	Sturgeon muscle	(32)
	PRRTRMMNGGR	Juice of cooked tuna	(38)
	KQSESHFVDAQPEQQQR	Simulated gastrointestinal digestion of extruded adzuki bean protein	(39)
	MSCP	*Chanos chanos*	(40)
	VVNEGEAHVELVGPKGNKETLEYES, AMPVNNPQIHDFFL	Beans (*Phaseolus vulgaris* var. pint)	(41)
	WNLNP	OPEH (*Crassostrea hongkongensis*)	(42)
Antimicrobial peptides	Turgencin A	Arctic sea squirt *Synoicum turgens*	(43)
	Myticusin-beta	Mytilus coruscus	(44)
	Temporin-1CEh	Rana chensinensis	(45)
	EQLTK	Bovine α-L A	(46)
	ISGLIYEETR, IGNGGELPR, ILVLQSNQIR	*Saccharina longicruris*	(47)
	cNK-2(RRQRSICKQLLKKLRQQLSDALQNNDD)	Chicken NK-lysin	(48)
	Clavanin-MO (FLPIIVFQFLGKIIHHVGNFVHGFSHVF-NH_2_)	Hemocytes of marine tunicates	(48)
	Phylloseptin-PV1	*Phyllomedusa vaillantii*	(49)
	GDVIAIR	Chia seed	(50)
	TSKYR, STVLTSKYR, TSKYR	Human hemoglobin: active peptide α137-141	(51)
	AGLAPYKLKPIA	Ovotransferrin	(52)
	YPWTQR, ITMIAPSAF, DSYEHGGEP, VVSGPYIVY	Egg yolk	(53)
Antioxidant active peptides	GGAW	Octopus	(54)
	JPHT-2	Jellyfish	(31)
	WSVPQPK	Human β-CN	(55)
	VPP, IPP	Whey protein concentrate (WPC)	(56)
	GAPGPQMV	Skipjack tuna (*K. pelamis*) bones	(57)
	GPGGFI	*N. septentrionalis* skin	(58)
	SMRKPPG	Peony (*P. suffruticos*) seed	(59)
	YFPH	*Limanda aspera*	(60)
	GFPGRLDHWCASE	Flaxseed (*Linum usitatissimum*)	(61)
	TSSSLNMAVRGGLTR, STTVGLGISMRSASVR	Finger millet (*Eleusine coracanac*) protein hydrolysate	(62)
	VECYGPNRPQF	Algae (*Chlorella vulgaris*) protein waste	(63)
	IDHY, VVER	Water-soluble protein (*Gracilariopsis chorda*)	(64)
	VLPVPQK	Milk	(65)
Anticancer active peptides	Callyaerins A-F, Callyaerins H	*Callyspongia aerizusa*	(66)
	Bowman-Birk-type PI	*Phaseolus acutifolius*	(67)
	Homophymine A	Marine sponge *Homophymia* sp.	(68)
	FIMGPY	Skate (*Raja porosa*) cartilage protein hydrolysate	(69)
Antihypertensive peptides	IVDR, WYK, VASVI	Paralichthys olivaceus	(70)
	VHVV	Soybean	(71)
	ERYPIL, VFKGL, WEKAFKDED, QAMPFRVTEQE	Egg white hydrolysate	(72)
	DGVVYY	Seed meal of tomato	(73)
	BCH, BCH-III	Chicken blood	(74)
	PPL, PAP, AAP	Iberian dry-cured ham	(75)
Neuroactive peptides	Doppelganger-related peptides	Cone snail toxins	(76)
	Arginine vasopressin	Hypothalamus	(77)
	Glucagon-like peptide-1	Proglucagon derived peptide	(78)
	Human urotensin-II	Central nervous system	(79)

The antioxidant effect of BAPs can slow down or prevent cellular damage (26). With the disturbance of the prevailing environment, oxidative stress reactions occur, resulting in the release of free radicals, which may contribute to health issues, including cancer, cardiovascular, and other diseases (27). These peptides primarily consist of 5-16 hydrophobic amino acids (27). They typically include tyrosine, whose phenolic side chain serves as an important scavenger of free radicals (28). Hydrophobic amino acids can increase the penetration rate of peptides to cell membranes, and enhance the ability of peptides to reach mitochondria, which is one of the main sites of free radical production (29, 30). An important feature of the antioxidant activity of BAPs is their hydrophobicity. It helps protect the polyunsaturated fatty acids and other lipophilic targets from oxidation (29, 30). Teng et al. (31) reported that jellyfish peptides (JPHT-2) were effective antioxidants which could scavenge free radicals. The peptides enhanced the levels of superoxide dismutase (SOD) and inhibited oxidative damage by H_2_O_2_. Gao et al. (32) reported a new anti-inflammatory peptide from sturgeon muscle, and found that it can effectively inhibit the release of NO, IL-6 and IL-1β, increase the SOD activity in the LPS-induced RAW264.7 cells, and down-regulate MAPK pathway. Zhou et al. (33) described that milk casein-derived peptide OEPVL could regulate the release of nitric oxide (NO) and the production of cytokines IL-4, IL-10, IFN-γ, and TNF-α *in vivo*, thereby achieving the purpose of inhibiting LPS-induced inflammation.

## 3 Anti-inflammation of BAPs

**Figure 1:**
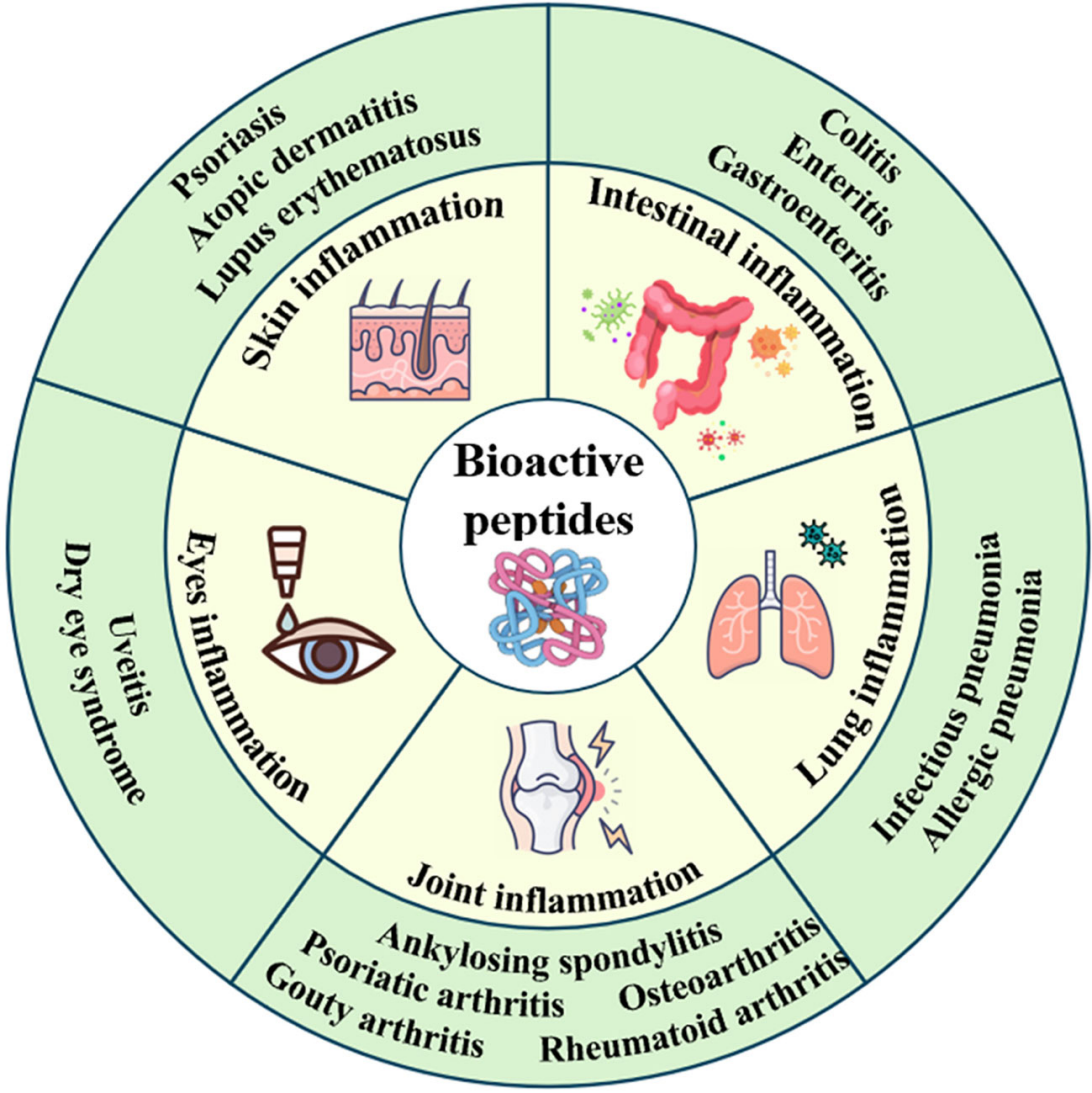
Scheme of the treatment of organ inflammation by BAPs.

As infection affects or damages different organs within the body, an inflammatory response occurs to combat infection, address injury, and facilitate self-repair. However, inflammatory factors can attack cells, leading to cell death, reduced cellular metabolism, and compromised immune function (3). BAPs can treat skin, intestine, lung, joint and eye inflammation, etc (**Figure 1**). BAPs can regulate the inflammatory pathways, the levels of cytokines or gut microbiota, and alleviate oxidative stress (**Table 2**).

**Table 2:** Application of BAPs in the treatment of inflammation of various organs.

Organ	Peptide names	Disease type	Peptide activity	Reference
Skin	LKEKK	Psoriasis	↑ IL-10, IFN-γ ↓ IL-17	(80)
	MHP1-AcN	Psoriasis	↓ IL-6, IL-23, IL-17A	(81)
	AES16-2M	Atopic dermatitis	↓ CD4 T cells ↓ TSLP	(82)
	TPS240	Atopic dermatitis	Inhibition of NF-κB and STAT3 activation	(83)
	AMP-IBP5	Atopic dermatitis	↑ TJ barrier function	(84)
	ARA290	Systemic lupus erythematosus	↓ IL-6, MCP-1, TNF-α ↑ TGF-β Suppressing the level of serum ANAs and anti-dsDNA autoantibodies Inhibiting the inflammatory activation of macrophages Promoting the phagocytic function of macrophages	(85)
Intestinal	rVIPa	Colitis	↓ TNF-α, MPO activity, serum endotoxin, TLR4 ↑ IL-10 ↑ occluding, ZO-1, NF-κB p65, IκBα	(86)
	R7I	Intestinal inflammation	Inhibition of TLR4 and NF-κB expression ↑ SOD and GSH-PX ↓ MDA	(87)
	MOP	Colitis	Inhibiting JAK-STAT pathway’s activation Regulating gut microbiota and its metabolites	(88)
	TBP	Ulcerative colitis	↑ SOD and GSH-Px ↓ LPS, IL-6, TNF-α ↑ Gene expression of TJ protein ↑ SCFAs Restoring intestinal flora	(89)
	Cecropin A (1-8)-LL37 (17-30)	Intestinal inflammation	↓ TNF-α, IL-6, IFN-γ ↓ Apoptosis ↓ Markers of jejunal epithelial barrier function	(90)
Lung	PS1-2	Fungal pneumonia	↓ Activity of TLR-2 ↓ TNF-α	(91)
	7-amino acid peptide (7P), (Gly-Gln-Thr-Tyr-Thr-Ser-Gly)	Allergic lung inflammation	↓ Airway hyperresponsiveness ↓ Airway inflammation ↓ Th2 responses	(92)
	IDR-1002	Pneumonia	↓ IL-6, TNF-α	(93)
	Hydrostatin-SN1	Acute lung injury	↓ TNF-α, IL-6, IL-1β	(94)
Joint	AKP	Osteoarthritis	↓ HIF-2α and downstream genes	(95)
	AESIS-1	Rheumatoid arthritis	Downregulation of STAT3 signaling	(96)
	KPs	Adjuvant-induced arthritis	Inhibiting IL-1β-related inflammation and MMPs production	(97)
	GLPP	Rheumatoid arthritis	↓ TNF-α, IL-1β, IL-6, MMPs, BCL-2, OPN, β-Catenin, HIF-1α ↑ Bax Inhibiting NF-κB and MAPK signaling pathways	(98)
	IQW	Ankylosing spondylitis	↓ IL-6, IL-1β, TNF-α ↑ CAT, GSH-PX, SOD	(99)
	Alamandine	Rheumatoid arthritis	↓ IL-6, IL-23 and IFN-γ mRNA expression ↓ TNF-α, IL-6, IL-17 ↑ IL-10	(100)
Eyes	R9-SOCS1-KIR	Uveitis	Inhibiting nuclear factor κB and p-p38 pathways	(101)
	WP-17	Uveitis	Inhibition of NF-κB pathway activation	(102)
	TSP	Dry eye disease	Regulating Bax/Bcl-2 signal pathway Inhibiting iNOS and COX-2 Moderating ROS/Nrf2/HO-1 axis Apoptosis inhibiting	(103)
Others	P140	Periodontitis	↓ TNF-α, INF-γ ↓ Infiltration of activated lymphocytes	(104)
	Nal-P-113	Periodontitis	↓ IL-1β, TNF-α	(105)
	Bomidin	Periodontitis	Downregulation of MAPK and NF-κB signaling pathways Activation of Keap1/Nrf2 pathway	(106)

↑ and ↓ indicated increase and decrease, respectively.

### 3.1 Skin inflammation

The skin serves as a physical barrier between internal and external environments (107). Various factors can induce inflammatory responses in the skin, primarily due to immune dysregulation caused by internal diseases, infections, and allergic reactions. Skin inflammation is a primary manifestation of chronic autoimmune inflammatory diseases such as psoriasis, atopic dermatitis (AD), and lupus erythematosus (108). Approximately, 60 million people suffer from psoriasis, a chronic, systemic, immune-mediated inflammatory skin disease (109). As previously described, the synthetic peptide LKEKK (150-500 μg) combined with Aldara cream containing 5% imiquimod was applied to the ears of the imiquimod-induced psoriasis mouse model (80). After 6 days of treatment, the thickness of mouse ears was significantly reduced, indicating that the development of inflammation was effectively inhibited. Traditional medications for AD often yield unsatisfactory results. Lee et al. (83) reported a short peptide TPS240 and investigated its therapeutic effect in a DNCB-induced AD mouse model. The control group was treated with the same concentration of dexamethasone. Finally, it was found that the symptoms of AD in the TPS240 group were alleviated, and the skin damage was significantly restored by using 5 mg/kg TPS240. The body weight of mice treated with 5 mg/kg dexamethasone decreased and the organs contracted abnormally. TPS240 exerts its anti-AD effect by inhibiting the activation of NF-κB and STAT3, which is similar to dexamethasone and has no side effects. These results indicated that TPS240 would be a safe and effective drug for AD. Systemic lupus erythematosus (SLE) is an autoimmune disease that can promote chronic inflammation (110). It has been reported that the artificial peptide pConsensus, which blocks the PD-1/PD-1 ligand 1 pathway in untreated mice, promotes tolerance and inhibits SLE (111). Schall et al. (112) reported the peptide P140 could clear harmful T and B cells, and normalize the immune response in lupus-susceptible mice.

Additionally, due to the skin’s susceptibility to various injuries, wounds disrupt its environmental barrier, leading to a cascade of inflammatory responses. Controlling inflammation is crucial for maintaining skin health. Li et al. (113) demonstrated that the peptide OA-RD17 extracted from *Odorrana-andersonii* skin tissue could activate MAPK to promote macrophage proliferation and migration, block inflammation and propel wound healing by inhibiting NF-κB. OA-RD17 could accelerate the regeneration of full-thickness skin wounds in mice, showing that the repair rate of skin wounds was nearly 100%. At the same time, it had a certain repair effect on deep second-degree burns and isolated skin wounds of diabetic patients. OA-RD17 could up-regulate the expression of miR-632 and promote the regeneration of full-thickness skin wounds in rats, and the repair rate reached 92.4%. Therefore, BAPs with their antimicrobial and immune-modulating functions offer efficacious therapeutic approaches for wound healing and skin inflammation.

### 3.2 Intestinal inflammation

The intestine plays a crucial role in human health, serving as a site for digestion and nutrient absorption, and the largest organ of the immune system (114). The intestinal barrier is essential for separating the external environment from the host’s internal environment. As the intestine is exposed to pathogens or other toxic substances, inflammatory responses occur under the influence of harmful stimuli (115). Enteritis is a prevalent inflammatory bowel disease. So far, the main methods used clinically for enteritis treatment include drug therapy, dietary interventions and surgical treatment. However, the treatment outcomes are often unsatisfactory. Therefore, it is very important to find a better and safer treatment method. BAPs have immunomodulatory and anti-inflammatory effects, making it possible to effectively treat intestinal inflammation and provide a new treatment for enteritis. Zhi et al. (116) reported that walnut-derived peptide leucine-proline-phenylalanine (LPF) could promote the repair of the intestinal epithelial barrier, reduce levels of pro-inflammatory cytokines, and exert protective and restorative effects on DSS-induced colitis in mice. It was found that the number of apoptotic cells in the treatment group was significantly less than that in the DSS group. The percentages of reduction in the three groups of DSS + 50 mg/kg LPF, DSS + 100 mg/kg LPF, and DSS + 200 mg/kg LPF on the 10th day were 50.00%, 41.18%, and 57.35%, respectively. In addition, 16S rDNA sequencing results showed that 100 mg/kg LPF had a regulatory effect on the intestinal flora of colitis mice. Additionally, Rahabi et al. (117) reported that fish collagen peptide Naticol^®^Gut could also be used to treat colitis. It directly acts on macrophages, polarizing them into an anti-inflammatory, immunotolerant, and antioxidative phenotype through an MR-dependent mechanism. For enteritis, antibiotics are often used for treatment, but their long-term use can lead to increased antibiotic resistance, posing a significant challenge. Sun et al. (118) reported that AMP R7I with anti-proteolytic properties could reduce inflammatory factors and maintain intestinal barrier function. The histological examination of the intestine showed that the tissue structure in the 20 mg/kg R7I group was basically normalized with only a small amount of isolated epithelial cells, and R7I could restore the normal morphology of the intestine. In addition, this peptide plays a crucial role in the treatment of murine bacterial enteritis and is helpful in finding effective strategies for the treatment of enteritis.

### 3.3 Lung inflammation

Pneumonia is a prevalent respiratory illness that involves inflammation in the lungs (119). Its occurrence is associated with respiratory viruses, common gram-negative or gram-positive bacteria, and mycobacterium (120, 121). Pneumonia has a complex etiology, and traditional treatment methods mainly involve the use of antibiotics, which can effectively reduce the incidence and mortality of pneumonia. However, issues such as antibiotic resistance, low bioavailability, and strong side effects exist (92, 122). Therefore, there is a necessity to discover novel treatment approaches. BAPs as a novel therapeutic drug may have potential in the treatment of pneumonia. Zhao et al. (92) reported that 7-amino acid peptide (7P), as a synthetic analog peptide, could effectively reduce bronchial contraction, inhibit acute inflammatory cytokines (TNFα, IL-1β and IL-6) and Th2 cytokine responses (IL-5, IL-4 and IL-13), and has certain effects on relieving airway hyperresponsiveness, airway inflammation and Th2 response. The results inferred that 7P could reduce allergic lung inflammation. It made a new option for addressing allergic pulmonary inflammation. Additionally, peptide modification can also be employed to improve the therapeutic effects. Moreira et al. (123) pegylated the synthetic peptide LyeTx I-b derived from natural LyeTx I, and reported that pegylated LyeTx I-b exhibited significant therapeutic effects against multidrug-resistant *Acinetobacter baumannii*-induced pneumonia. LyeTx I-bPEG increased the anti-biofilm activity. At 16 μM and 32 μM, LyeTx I-bPEG reduced the carbapenem-resistant *Acinetobacter baumannii* biofilm by 33 ± 4% and 26 ± 8%, respectively, compared with untreated cells. Furthermore, Jin et al. (124) designed two derived peptides GHbK4R and GHb3K based on the maternal peptide GHb. Vancomycin reduced lung bacteria in mice to 7.8 × 10^7^ CFU/g, whereas GHb3K and GHbK4R decreased lung bacteria to 5.3 × 10^5^ and 5.4 × 10^5^ CFU/g. These results demonstrated that these peptides had significant therapeutic effects in a mouse model of acute pneumonia caused by *Staphylococcus aureus* infection. PS1-2 peptide is active against fluconazole-resistant *Candida albicans*, can inhibit the activity of TLR-2 and the expression of TNF-α, and has anti-fungal and anti-inflammatory functions for intratracheal infection induced by *Candida albicans* (91). However, there is limited research on the use of BAPs for the treatment of human pneumonia. It still needs a good strategy to treat pneumonia.

### 3.4 Joint inflammation

Arthritis is a common inflammatory disease which affects the joints and surrounding tissues. It can be acute or chronic, leading to joint pain, swelling and difficulty movement in severe cases. Arthritis has a high prevalence and encompasses various types, including osteoarthritis, rheumatoid arthritis, and psoriatic arthritis (125). Osteoarthritis is a progressive disease and a major cause of chronic disability (126). Peptides offer a new therapeutic approach for osteoarthritis. Wu et al. (127) validated that the anti-inflammatory capacity of skipjack tuna elastin peptides in a zebrafish model could inhibit the JAK2/STAT3 signaling pathway, suppress inflammation and protect cartilage. Rheumatoid arthritis is an autoimmune disease that can lead to joint and bone damage (128, 129). For rheumatoid arthritis, Kim et al. (96) reported that a synthetic peptide AESIS-1 could inhibit STAT3-mediated signaling by upregulating SOCS3 expression, resulting in the decrease of Th17 cells. Psoriatic arthritis is a chronic systemic inflammatory disease affecting the skin, joints, and tendons (130). Wixler et al. (131) discovered small splenic peptides (SSPs) in the spleen, which could target dendritic cells and transforming them into tolerant cells, thus differentiating naive CD4 cells into regulatory T cells expressing Foxp3. SSPs had anti-inflammatory effects *in vivo*, and restore peripheral tolerance, effectively inhibiting the development of psoriatic arthritis. In addition, ankylosing spondylitis and gouty arthritis could be treated by using BAPs. Ankylosing spondylitis is an immune-mediated chronic inflammatory rheumatic disease that most commonly affects the spine (132). Liu et al. (99) reported that BAPs IQW could treat mice with ankylosing spondylitis, delay disease progression, alleviate inflammation in the intervertebral joints, and reduce the concentration of pro-inflammatory factors. Gouty arthritis is caused by inflammation triggered by the deposition of urate crystals in the joints and surrounding tissues (108). Commonly used medications include colchicine, corticosteroids, NSAIDs, and adrenocorticotropic hormone, but these drugs have certain side effects such as nausea and gastrointestinal toxicity. Therefore, there is an urgent need to develop new drugs to treat gouty arthritis (133). Yan et al. (134) described that BAPs mastoparan M (Mast-M) extracted from wasp venom could inhibit the MAPK/NF-κB signaling pathway and reduce oxidative stress, thereby blocking the activation of the NLRP3 inflammasome and effectively treating gouty arthritis. Hence, BAPs have good therapeutic effect on joint inflammation.

### 3.5 Eyes inflammation

Eye inflammation is a common ocular condition that can occur from the surface of the eye to intraocular tissues (135). As threatened by inflammation, the eye tissues can sustain damage over the short or long term (136). The causes of eye inflammation are varied, including pathogen infections such as bacterial, fungal, and viral infections, as well as non-infectious factors like external environmental stimuli and allergic reactions (137). The treatment of eye inflammation mainly involves the use of anti-inflammatory drugs and antibiotics for medication or surgical methods. However, these approaches have certain drawbacks such as drug side effects and long recovery times. In recent years, more BAPs with therapeutic potential have emerged. Lu et al. (102) designed a peptide called WP-17, which targeted the toll-like receptor 4 (TLR4) to inhibit the activation of the NF-κB pathway. The highest dose of WP‐17 (10 μg/eye) strikingly decreased the protein levels of TNF‐α and IL‐6 in the aqueous humor of rats by 77.26% and 85.67%, respectively. WP-17 has shown promising therapeutic effects in rat uveitis. Similarly, Ho et al. (138) reported that a 29-mer peptide derived from pigment epithelium-derived factor could inhibit the expression of matrix metalloproteinase-9 and pro-inflammatory cytokines on murine dry eye. In addition, Zeng et al. (103) described that tilapia skin peptides (TSP) impeded the generation and development of dry eye disease via inhibition of apoptosis (19.4%), inflammation, and oxidative stress.

### 3.6 Other inflammation

The oral cavity is an important part of the human body and serves as the starting point of the digestive system. The oral cavity harbors a rich microbial population, constituting the second abundant microbial community in the human body after the gut, with over 700 identified oral microbial species (139, 140). Disruption of the oral microbiota can lead to an increase in local T_H_17 cells, which are associated with oral immunity and inflammation (141). Dysbiosis of the oral microbiota can lead to periodontitis, a common oral disease caused by pathogens invading the periodontal tissues such as the gums (142, 143). BAPs can inhibit bacterial growth and reduce inflammation. Akiyama et al. (104) reported the role of peptide P140 in a mouse model of periodontitis, and found that treatment with P140 effectively alleviated inflammation in gingival tissues, reduced lymphocyte infiltration, and lowered the expression of pro-inflammatory mediators. In addition, liver injury can also be treated by bioactive peptides. Zhu et al. (144) described a peptide HEPFYGNEGALR isolated and identified from *Apostichopus japonicus*. This peptide can activate the Nrf2/HO-1 pathway, block the nuclear translocation of NF-κB, alleviate oxidative stress and inflammation, and alleviate acute alcoholic liver injury caused by excessive alcohol intake. Besides, BAPs have a certain ability in the treatment of myocarditis. Cortistatin is a small molecule bioactive peptide (145). Delgado-Maroto et al. (146) reported the therapeutic effect of cortistatin in experimental autoimmune myocarditis, and found that it could inhibit the inflammatory response driven by cardiomyogenic T cells.

### 3.7 Clinical application of BAPs

Peptides and peptidomimetics are emerging as an important class of clinic therapeutics (147). However, their application is hindered by their poor stability, short half-life, and low retention rate (148). It was reported that cyclic peptide structures had high topological flexibility, and their shape changes without transforming the amino acid composition sequence could not alter their properties (149). Therefore, molecular grafting is a good choice. It has been demonstrated that bradykinin antagonists were conjugated onto cyclic peptide scaffolds for the inflammation treatment (150). And sustained-release peptide analogues can be used for clinical treatment (151). BAPs are widely used to regulate inflammatory pathways and inflammatory factors to treat inflammation in clinics. Brimapitide (XG-102), a peptide bound to the N-terminal sequence of c-Jun, inhibits JNK by competing with endogenous c-Jun. In this way, it suppresses inflammation caused by JNK. This drug is currently under Phase III (149). Thymosin alpha-1 is an immunostimulatory peptide. It can regulate the immune system, enhance T cell function, inhibit the release of pro-inflammatory cytokines, and promote the production of anti-inflammatory cytokines (152). It is clinically used to treat hepatitis B (153). Since one century ago, more than 80 peptide drugs have reached the market for a wide range of diseases, including diabetes, cancer, osteoporosis, multiple sclerosis, HIV infection and chronic pain (154). However, there are still few peptides as clinical drugs for the treatment of inflammation.

## 4 Anti-inflammatory mechanism of BAPs

### 4.1 Regulation of the release of inflammatory mediators

Chemical substances released by cells or produced by body fluids during the inflammatory process, which participate in or cause the inflammatory reaction, are referred to as inflammatory mediators. They mainly include prostaglandins, NO, cytokines like interleukins (IL) (e.g., IL-1β, 2, 6, and 8), chemokines, etc. (155). As activated through toll-like receptors (TLR), these innate immune cells induce the release of IL-6 and TNF-α, along with transforming growth factor-β, which facilitates cell proliferation (156). The NF-κB and MAPK are also key pro-inflammatory intermediaries that are produced after TLR activation (157). Cytokines are low molecular weight glycoproteins produced and secreted by different cells, which can regulate the proliferation and differentiation of immune cells (158). They can be divided into two major categories: pro-inflammatory and anti-inflammatory factors. Pro-inflammatory factors such as IL-1β and TNF-α further induce the inflammatory response, while anti-inflammatory factors such as IL-10 can promote the resolution of the inflammatory response (159). Many studies show that BAPs can regulate the release of inflammatory mediators. Tornatore et al. (157) isolated four peptides from eggs white and these peptides exhibited anti-inflammatory activities in colitis mice by inhibiting the production of TNF-α and IL-6 as well as reducing the mRNA-expressions TNF-α, IL-6, IL17, IL-1β, IFN-γ, and MCP-1. Xing et al. (160) reported that bovine bone gelatin peptides could alleviate the additional secretion of inflammatory factors IL-6, NO, and TNF-α induced by lipopolysaccharide (LPS) in RAW264.7 cells to mitigate DSS-induced colitis. Cresti et al. (161) conducted efficacy studies on the synthetic peptide SET-M33 targeting gram-negative bacteria by using an LPS-induced pneumonia model. They found that the peptide effectively reduced the production of pro-inflammatory cytokines KC, MIP-1α, IP-10, MCP-1, and TNF-α.

### 4.2 Regulation of inflammatory signaling pathways

**Figure 2:**
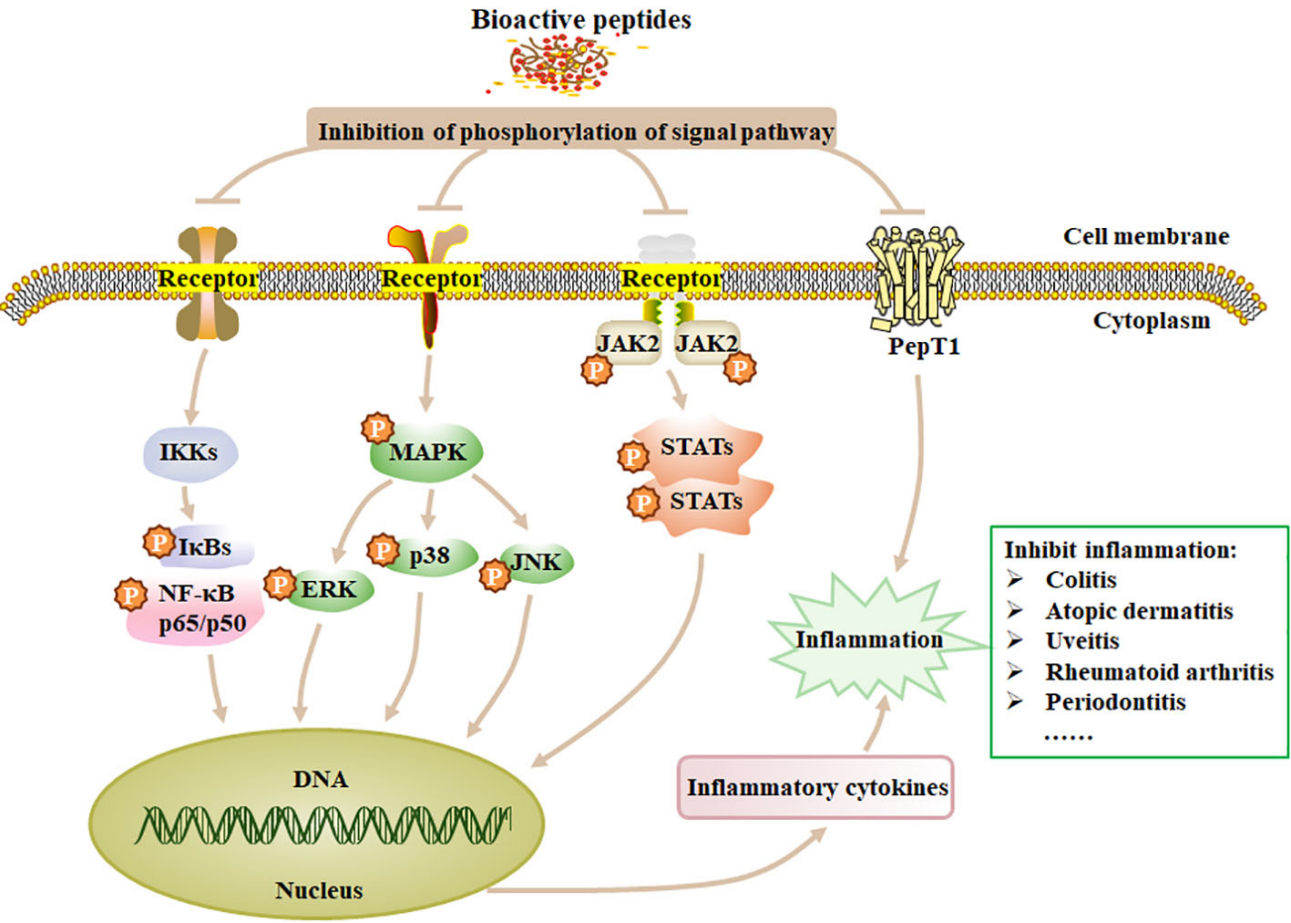
The mechanism of anti-inflammation of BAPs. Treatment of inflammation by modulating the four signaling pathways, such as NF-κB, MAPK, JAK and STATs. p, phosphorylation; Ikks, inhibitor of kappa B kinase. Adapted from previous reports (167).

Inducers like LPS can stimulate and activate key proteins or genes involved in cellular signaling pathways such as NF-κB pathway (162) and MAPK pathway (163). The anti-inflammatory peptides inhibit cell inflammatory responses mainly through the MAPK and NF-κB pathways. NF-κB pathway is the most important way to regulate the transcription of pro-inflammatory cytokines such as IL-6, IL-1β and TNF-α, and also plays a vital role in the expressions of inducible nitric oxide synthase (iNOS) and COX-2 (164). NF-κB is a family of transcription factor proteins, including five subunits: p65 (RelA), p50, p52, Rel, and RelB. After dimer p65/p50 is released into the cytosol, it can be translocated into the nucleus and initiates target gene transcription for pro-inflammatory factors, causing inflammation (164, 165). MAPK can regulate many cellular activities, including proliferation, differentiation, death and immune response. The stimulus and MAP3K phosphorylation can mediate the phosphorylation of the downstream MAP2K and MAPK, which contain three subfamilies: p38, extracellular signal-regulated kinases (ERK1 and ERK2), and c-Jun N-terminal kinase (JNK). In unstimulated cells, JNK mainly exists in the cytoplasm and partly distributes in the nucleus. After being stimulated, JNK accumulates in the nucleus and causes the corresponding gene (IL-1 and TNF-α) expression, resulting in an inflammatory response (166). BAPs inhibit the expression of inflammatory genes by blocking NF-κB and MAPK signaling pathways (**Figure 2**). The JAK-STAT pathway is also important for inflammatory response, which can regulate hematopoietic cell development and inflammatory cytokines (167). Phosphorylation of JAK and STATs can form the dimer translocated to the nucleus (168). In addition, the peptide transporter PepT1 can transport small BAPs to the bloodstream. Therefore, the role of PepT1 is vital to the bioactivity of BAPs (167). Chei et al. (169) described that acid-hydrolyzed silk peptide (SP) inhibited LPS-induced inflammation by modulating the TLR4 signaling pathway, while clam peptide MMV2 reduced the mRNA levels of inflammation-related genes induced by LPS in adult zebrafish (170). Formyl peptide receptors (FPRs), members of the GPCR family with seven transmembrane domains (171), play important roles in antimicrobial host defence mechanisms. FPRs recognize formylated peptides, non-formylated peptides, synthetic small molecules, and formyl analogs from bacteria and mitochondria to regulate inflammatory responses that lead to chemotaxis, degranulation, and oxidative bursts (172). Jin et al. (173) reported that VLATSGPG (VLA), a DPP-IV inhibitory peptide isolated from the skin of *Salmo Salar*, could inhibit the activation of PERK through the AKT signaling pathway, and increase the expression of IκBα mRNA through the PERK/IκBα pathway, leading to blocking the activation of NF-κB p65 and further cell inflammation. Tsuruki et al. (174) isolated some immunostimulating peptides from soy protein, which had specific binding sites on mouse or human macrophages and could stimulate their phagocytic activity.

### 4.3 Regulation of reduced oxidative stress response

**Figure 3:**
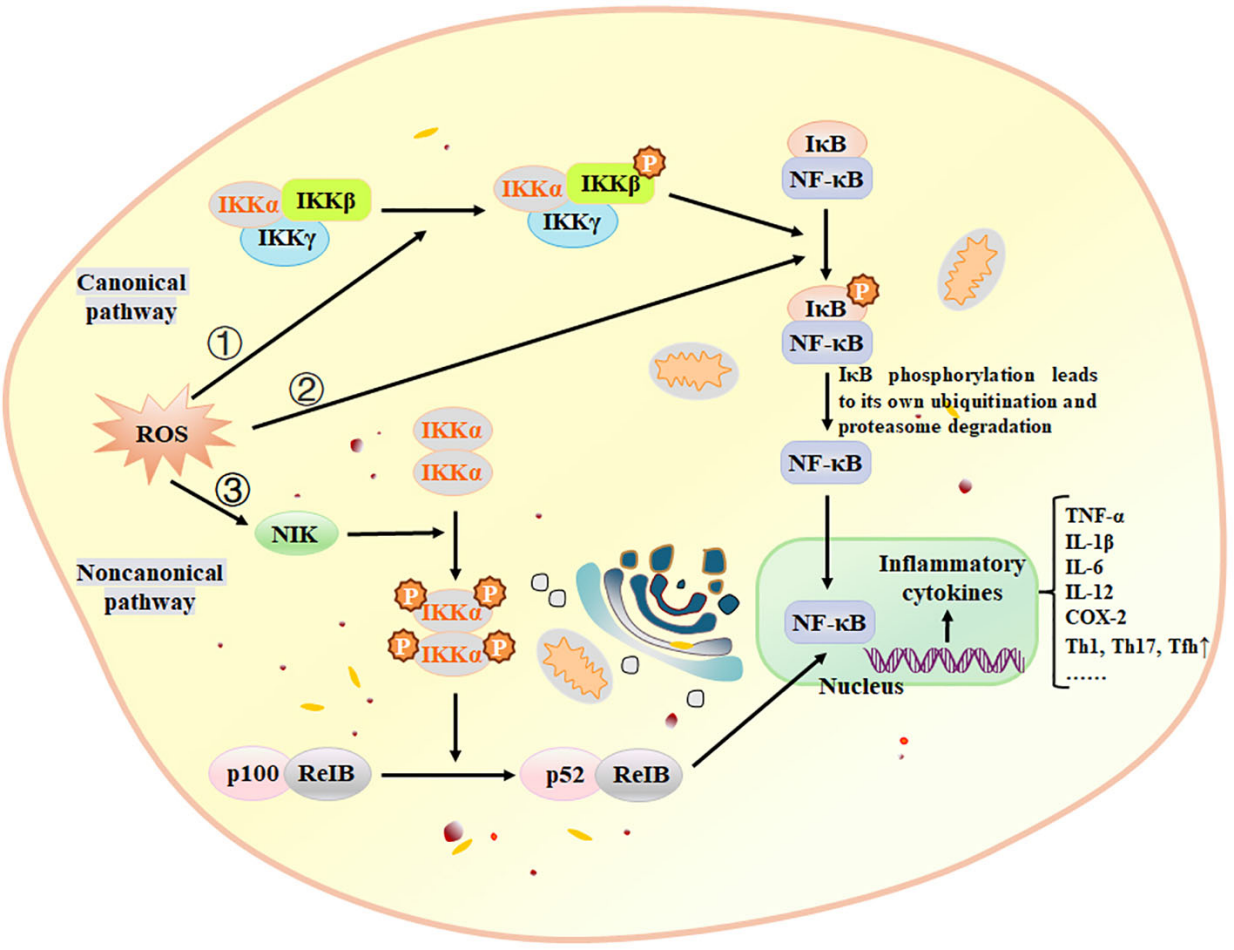
ROS activate NF-κB through three pathways. ① Canonical pathway: ROS activates the IKK complex, phosphorylating IκBα. Phosphorylation leads to ubiquitination and proteasomal degradation of IκBα, resulting in nuclear translocation of the NF-κB complex and gene expression through high-affinity binding to κB components, ② ROS directly phosphorylate IκBα, subsequently following the same pathway as the canonical pathway, ③ Noncanonical pathway: NIK is activated by ROS through inhibition of phosphatases and oxidation of cysteine residues. The NF-κB activation pathway relies on IKKα and activates the p52/RelB complex by triggering proteolytic cleavage of the p52/p100 precursor. IKK, IκB kinase; NIK, NF-κB-inducing kinase. Adapted from previous reports (181).

Oxidative stress is a significant pathological factor that contributes to various inflammatory diseases. Inflammatory responses trigger the excessive generation of reactive oxygen species (ROS) within cells, disrupting the body’s free radical metabolism and leading to oxidative stress. Moreover, during oxidative metabolism, excessive ROS can attack cells or tissues, causing structural and functional damage and exacerbating inflammatory reactions (175, 176). BAPs can reduce the generation of ROS. Lee et al. (177) isolated the peptide PPY1 from *Pyropia yezoensis*, and stated that PPY1 significantly decreased the ROS levels in LPS-induced macrophages. Oxidative stress and inflammation are closely related, which can elucidate why NF-κB is the initial mammalian transcription factor to be influenced by oxidation (178). NF-κB plays a crucial role in mediating inflammatory responses and is regulated by various mediators, including H_2_O_2_ and ROS (178). ROS can modulate NF-κB through both the Canonical and Noncanonical pathways (**Figure 3**). Malondialdehyde (MDA) and glutathione (GSH) are important markers of oxidative stress. MDA is the final product of ROS-induced lipid peroxidation, while GSH is an intracellular antioxidant that protects cells from oxidative stress damage. Peng et al. (54) identified an active peptide, GGAW, which exhibits excellent antioxidant functionality. This peptide effectively reduces the production of ROS, MDA and lactate dehydrogenase (LDH), and increases the activity of SOD and glutathione peroxidase (GSH-PX). Consequently, it enhances cell viability and protects IEC-6 cells from H_2_O_2_-induced oxidative damage. The Kelch-like ECH-associated protein 1-(Keap1) Nrf2-antioxidant response element is the main antioxidant signaling pathway that prevents oxidative stress and helps maintain the optimum redox steady state *in vivo* (179). Hence, the Nrf2 antioxidant signaling pathway can be stimulated to suppress oxidative stress within the body (167). Fernando et al. (180) reported that AMVDAIAR, a peptide isolated from pepsin hydrolysate of krill enhanced antioxidant enzymes SOD, CAT and GPx, thereby suppressing the oxidative stress in H_2_O_2_-induced hepatocytes and increasing the expression of Nrf2.

## 5 Conclusions and prospects

BAPs are widely employed in the treatment of inflammation. This review summarizes the therapeutic effects of BAPs on various inflammatory diseases such as pulmonary, gastrointestinal, dermatological, arthritic, oral and ocular inflammations. It also outlines the anti-inflammatory mechanisms of action of BAPs, which include modulation of inflammatory mediators’ release, regulation of inflammatory signaling pathways (NF-κB, MAPK, and JAK-STAT), and reduction of oxidative stress reactions to influence the development of inflammation.

BAPs have promising prospects for the preparation of anti-inflammatory drugs. However, BAPs are commonly implicated with several challenges, encompassing a short half-life, susceptibility to proteases, instability, potential toxicities, and other processing-related issues. Attempts can be made to modify or transform the BAPs, such as by attaching metal ions, targeting groups or nanomaterials to maximize their effectiveness. However, before using BAPs to treat various inflammatory diseases, more experiments are needed to obtain additional data on dosages, pharmacodynamics and pharmacokinetics. Studies should also investigate the differential effects of BAPs on different populations to better understand their efficacy. Furthermore, the anti-inflammatory mechanisms of various types of BAPs require investigation to ensure their safety in clinical applications. Additionally, many peptides face challenges in maintaining stability and functional activity *in vivo* due to inherent limitations of amino acids. BAPs can be encapsulated within nanoparticles to improve their stability. Future efforts should concentrate on finding more methods to overcome these challenges to maximize the efficacy of BAPs. In conclusion, BAPs hold great promise as potential inflammatory therapy. Further research and clinical data are necessary to support their widespread and safe application.
